# Theoretical exploring of potential mechanisms of antithrombotic ingredients in danshen-chishao herb-pair by network pharmacological study, molecular docking and zebrafish models

**DOI:** 10.1186/s13020-024-00970-6

**Published:** 2024-07-16

**Authors:** Chang Rao, Ruixue Hu, Yongxin Hu, Yan Jiang, Xu Zou, Huilan Tang, Xing Chen, Xiaoli He, Guang Hu

**Affiliations:** 1 School of Pharmacy and Bioengineering, Chongqing University of Technology, Chongqing, 400054 China; 2 Chongqing Institute for Food and Drug Control, Chongqing, 401121 China; 3 Department of Pharmacy, Women and Children’s Hospital, Chongqing Medical University, Chongqing, 401147 China; 4 Children’s Hospital of Yongchuan District, Chongqing, 402160 China

**Keywords:** Salvia miltiorrhiza, Radix Paeoniae Rubra, Salvianolic acid A, Paeoniflorin, Zebrafish, Network pharmacology, Molecular docking, Antithrombotic effect

## Abstract

**Background:**

Salvia miltiorrhiza (Danshen, DS) and Radix Paeoniae Rubra (Chishao, CS) herbal pair (DS-CS) is a famous traditional Chinese combination which has been used as antithrombotic formular for centuries. However, there is still lack of sufficient scientific evidence to illustrate its underlying mechanisms. The purpose of this study is to investigate the antithrombotic effects of DS-CS extract in zebrafish and explore its possible mechanism of action.

**Methods:**

The quality of traditional Chinese medicines DS and CS granules was evaluated using High Performance Liquid Chromatography (HPLC). Subsequently, the therapeutic effect of the DS-CS combination and its components, Salvianolic Acid A (SAA) and Paeoniflorin (PF), in various concentrations on thrombosis was experimentally validated. Moreover, the interaction between DS-CS and the thrombosis disease targets was analyzed through network pharmacology, predicting the potential antithrombotic mechanism of DS-CS. Molecular docking and in vivo zebrafish experiments were conducted to validate the predicted targets, with qRT-PCR utilized for target validation.

**Results:**

DS-CS exhibited anti-thrombotic effect in zebrafish with concentrations ranging from 25 to 300 μg/mL. The co-administration of PF and SAA at 25 μg/mL each revealed a synergistic antithrombotic effect exceeding that of individual components when contrasted with PHZ treatment. Protein–protein interaction (PPI) analysis identified key genes, including Albumin (ALB), Proto-oncogene tyro-sine-protein kinase Src (SRC), Matrix metalloproteinase-9 (MMP9), Caspase-3 (CASP3), Epidermal growth factor receptor (EGFR), Fibroblast growth factor 2 (FGF2), Vascular endothelial growth factor receptor 2 (KDR), Matrix metalloprotein-ase-2(MMP2), Thrombin (F2), and Coagulation factor Xa (F10), associated with the antithrombotic action of PF and SAA. Furthermore, KEGG pathway analysis indicated involvement of lipid metabolism and atherosclerosis pathways. Molecular docking revealed strong binding of PF and SAA to pivotal hub genes, such as SRC, EGFR, and F10. The experimental findings demonstrated that DS-CS could upregulate the mRNA expression levels of EGFR while inhibiting F10 and SRC mRNA levels, thereby ameliorating thrombotic conditions.

**Conclusion:**

This research provided valuable insights into the potential mechanisms underlying the antithrombotic activity of DS-CS. Our findings suggested that PF and SAA could be the key active ingredients responsible for this activity. The antithrombotic effects of DS-CS appeared to be mediated through the regulation of mRNA expression of SRC, EGFR, and F10. These results enhanced our understanding of DS-CS's therapeutic potential and lay the groundwork for future studies to further elucidate its mechanisms of action.

## Introduction

Thrombosis, which plays an important role in cardiovascular disease, seriously threatens human health and life [1, 2]. Currently, the most commonly used antithrombotic drugs are aspirin, warfarin and heparin, however, they are accompanied by adverse reactions such as bleeding and drug resistance [3, 4]. Thrombosis refers to complex pathological conditions which require combinational therapies that can act on multiple biological targets [5]. Herbal medicines with antithrombotic properties have a long history of treating CVDs (e.g., arteriosclerosis, ischemic heart disease, stroke) by preventing thrombosis [6].

Compared with single herb and complicated formula, herbal pair is an ideal re-search object for the study of interaction between components. According to TCM compatibility theory, herbs may have limited effect when used alone but show enhanced or synergistic results with particular combinations [7]. Formula is the main type applied clinically, which contains multiple Chinese medicines following the rules of compatibility theory, but most of them contains too many components which is difficult to illustrate the underlying mechanism of action. Herbal pair not only reflect the synergistic effect of TCM, but also simplify the complex formula [7], surpassing the insights obtained from individual herbs or complex multi-herb formulas. Furthermore, some TCM herbal pair is further investigated and simplified into compound pair which presents some of the same pharmacological effect as the herbal pair, in the purpose of elucidating the mechanism of action involved.

Salviae miltiorrhiza (Danshen, DS) is a well-known herb in TCM and the active ingredient in DS has been shown to exert a variety of pharmacological effects, such as anti-inflammatory, antioxidant, antiapoptotic, and neuroprotective activities [8]. Radix Paeoniae Rubra (Chishao, CS), another herb used in TCM belonging to Ranunculaceae exhibits significant therapeutic effect on cardiovascular disease [9]. In TCM, DS and CS combination (DS-CS) stands out as a typical and frequently utilized pairing [10, 11]. However, the existing scientific literature indicates a limited number of reports dedicated to the mechanistic study of DS-CS.

Zebrafish (Danio rerio) have been widely used as a flexible model organism in the research of human diseases and related pathology, especially in the field of cardio-vascular diseases [12]. Compared to the conventional mammal in vivo models, which are usually laborious, costly, and time consuming [13], the zebrafish model organism has a large number of advantages including high fecundity, small size, rapid development, and rapid generation time [13]. In addition, the transparency of zebrafish embryos enables nonintrusive visualization of organs and biological processes in vivo with a high resolution. Furthermore, zebrafish thrombocytes are homologous to mammalian platelets, and the hemostatic mechanism of zebrafish is similar to that of human [14]. All these characteristics make zebrafish an excellent model to study cardiovascular disease and the mechanism of drug action [15, 16].

Network pharmacology is a research method that uses a database to analyze and predict the targets of multiple compounds. It can analyze the correspondence between multiple drugs and targets simultaneously and use the form of network diagrams to show the relationships [17]. Network pharmacology has the research approach of multiple components and targets, which is consistent with the integrated characteristics of TCM [18].

In our investigation, we confirmed the antithrombotic efficacy of a combination of SAA and PF, two representative compounds derived from DS and CS, respectively. They were administered at concentrations of 25 μg/mL, with an equal 1:1 ratio between the two compounds. Additionally, we conducted a network pharmacological analysis, molecular docking and In vivo experiment to investigate potential target molecules. These findings are anticipated to furnish valuable data elucidating the synergistic interactions between compounds within DS-CS and shedding light on the putative mechanisms of action.

## Materials and methods

### Chemicals and reagents

DS granules (Lot: 0422005501) and CS granules (Lot: 0422002211) were purchased from Kangrentang Co., Ltd. (Beijing, China). SAA was purchased from herbsubstance Co., Ltd. (Chengdu, China). Salvianolic acid B (SAB) was purchased from shyuanye Co., Ltd. (Shanghai, China). PF was purchased from solarbio Co., Ltd. (Beijing, China). N-Phenylthiourea (PTU), phenylhydrazine (PHZ), acetylsalicylic acid (aspirin, ASP), ethyl 3-aminobenzoate methanesulfonate (MS-222) and 3,3’-dimethoxybenzidine (O-dianisidine) were purchased from Shanghai Aladdin Biochemical Technology Co., Ltd. (Shanghai, China). Dimethyl sulfoxide (DMSO), methanol, acetonitrile and phosphoric acid were purchased from Shanghai Macklin Biochemical Co., Ltd. (Shanghai, China). Hydrogen peroxide (30%, H_2_O_2_) (AR) was purchased from Chengdu Kelong Chemical Co., Ltd. (Chengdu, China). Sodium acetate anhydrous (CH_3_COONa) (AR) was purchased from Tianjin Zhiyuan Chemical Reagent Co., Ltd. (Tianjin, China). Ethanol (AR) was purchased from Chongqing Chuandong Chemical (Group) Co., Ltd. (Chongqing, China). Other regular reagents for the daily maintenance of zebrafish system were purchased from Wuhan Tianzhengyuan Biological Technology Co., Ltd. (Wuhan, China). PCR primers were synthesized by Sangon Biotech. (Shanghai, China). Real-time polymerase chain reaction (real-time PCR) kits were purchased from TaKaRa Biotechnology Co., Ltd. (Beijing, China). All buffers and other reagents were of the highest purity commercially available.

### Sample preparations

The reference compounds, SAB and PF, were subjected to precise weighing, followed by dissolution in methanol to yield reference compound solutions with concentrations of 22.4 μg/mL and 21.8 μg/mL, respectively. These solutions were rigorously stored at a controlled temperature of 4 °C prior to their deployment in analytical processes.

Three distinct pairs of 1 g each, comprising DS and CS granules (with ratios of DS to CS at 1:0, 1:1, and 0:1), were individually prepared: Initially, 1.0 g of the granule pair was dispersed in 20 mL of 80% methanol within a covered 50 mL conical flask. Subsequently, the mixture underwent extraction in an ultrasonic container for approximately 20 min. The resultant extract solutions were subjected to sequential filtration, followed by their combination and subsequent filtration through a 0.22 μm membrane filter in preparation for subsequent HPLC analysis.

DS-CS extract for the treatment to zebrafish was prepared as follows: DS and CS granules were finely ground into powder and dissolved in ultrapure water at a 1:1 ratio, resulting in a final concentration of approximately 10 mg/mL. These extract solutions underwent centrifugation (4 °C, 5000 × g) for 15 min, with the process repeated twice. The supernatant was collected and subsequently filtered through 0.45 μm and 0.22 μm membrane filters. The filtered solutions were stored at −80 °C until further use.

A stock solution of ASP (300 μg/mL) was prepared in dimethyl sulfoxide (DMSO). Similarly, stock solutions of SAA and PF were prepared in DMSO at a concentration of approximately 10 mg/mL and then diluted with water to the desired concentration for zebrafish assays.

### HPLC analysis

HPLC analysis was conducted at a wavelength of 230 nm using a Shimadzu High Performance Liquid Chromatography LC-20AT system. Chromatographic separation was achieved utilizing an InertSustain C18 analytical column (250 mm × 4.6 mm, 5 μm). The mobile phase, comprising 0.1% aqueous acetic acid (A) and acetonitrile (B), was delivered at a flow rate of 1 mL/min. The gradient program was programmed as follows: 0–13 min, 16% B; 13–20 min, 16%–18% B; 20–25 min, 18%–20% B; 25–40 min, 20%–23% B; 40–50 min, 23%–25% B; 50–60 min, 25% B. The injection volume for all samples was maintained at 10 μL.

### Zebrafish maintenance and embryo collection

Wild-type AB strain adult zebrafish (*Danio rerio*, 4 to 6 months old) were purchased from the Shanghai FishBio Co., Ltd. (China) and maintained in an automated fish housing system at 28.5 ± 0.5 °C under a 14:10 h light to dark cycle, and fed freshly hatched brine shrimps three times daily. Embryos were obtained from spawning adults in a breeding chamber overnight with a sex ratio of 2:1 (male to female) ac-cording to the standard zebrafish breeding protocol. The embryos were collected within 40 min after the light was switched on and rinsed in E3 medium at 28.5 ± 0.5 °C.

### The exposure experiment of zebrafish larvae

Zebrafish survival and hatching rates tests were was conducted using 1-day post-fertilization (dpf) developing zebrafish embryos. DS-CS extract was dissolved in E3 medium to achieve concentrations of 0, 100, 200, and 400 μg/mL. Stock solutions of SAA and PF were dissolved in E3 medium to achieve concentration of 0, 25, 50, 100 and 200 μg/mL. A 24-well plate was utilized, with each well containing 1 mL of E3 medium mixed with the respective compounds and 10 embryos. The embryos were closely monitored daily for any signs of abnormalities.

Throughout the experiments, zebrafish were exposed to water containing 0.2 mM PTU from 24 hpf. To induce thrombosis, the thrombus-inducing chemical, PHZ, was administered to the zebrafish. Zebrafish embryos (15 per well) were treated in 24-well plates, with three parallel wells designated for each treatment group. The control group received 0.2 mM PTU, while the model group was exposed to 1.5 μM PHZ and sample solutions, including aspirin at 25 μg/mL, DS-CS at various concentrations (12.5, 25, 50, 100, 200, and 300 μg/mL), and PF at 25 μg/mL combined with different concentrations of SAA (0–25 μg/mL). After incubating in an incubator at 28 °C for 48 h, all the incubation solutions were discarded and the zebrafish were stained with o-dianisidine dye liquor for 30 min in the dark at 28 °C. Then, the zebrafish were rap-idly washed by DMSO three times. The anti-thrombotic effects of the various treatment groups were assessed by observing and photographing thrombi in the heart of zebrafish larvae using an Olympus-BX43 upright fluorescence microscope equipped with cellSens Standard software. The dyeing area of heart (S) was quantified by Image-pro Plus 6.0. The antithrombotic effects of different groups were evaluated based on the following formula [19]:

1 $${\text{Thrombosis inhibition percentage }}\left( \% \right)\, = \,\left[ {{\text{S}}\left( {{\text{drug}}} \right){-}{\text{S}}\left( {{\text{model}}} \right)} \right]/\left[ {{\text{S}}\left( {{\text{control}}} \right){-}{\text{S}}\left( {{\text{model}}} \right)} \right]\, \times \,{1}00\%$$

### Data collection

To obtain comprehensive data for our study, we initiated the process by retrieving information on chemical constituents from the Traditional Chinese Medicine Systems Pharmacology Database (TCMSP) at http://tcmspw.com/tcmsp.php. Subsequently, we employed SwissTargetPrediction (http://www.swisstargetprediction.ch/) and PharmMapper (http://lilab-ecust.cn/pharmmapper/index.html) to predict the targets associated with these components. The identified constituents were linked, either directly or indirectly, to their corresponding human target genes via the UniProt Database (http://www.uniprot.org/).

Furthermore, we accessed the GeneCards, Online Mendelian Inheritance in Man (OMIM), PharmGKB, and DrugBank databases to gather information on targets related to thrombosis. By cross-referencing these databases with the targets associated with PF and SAA, we established a dataset that encompassed the shared factors related to thrombosis and our compounds of interest.

### Protein–Protein interaction (PPI) network construction

To unravel the interactions among therapeutic target genes and identify pivotal genes, we integrated the shared target genes into the Search Tool for the Retrieval of Interacting Genes/Proteins (STRING) database, version 11.0 (https://string-db.org/) [20]. We specified "Homo sapiens" as the organism and set the confidence parameter to the high level (0.400) to procure PPI data. Hub genes were identified through topo-logical analysis. Visualization of the PPI network and subsequent topology analysis were executed using Cytoscape software. Combined with PPI network analysis, the targets unrelated to thrombosis were screened out, and PF and SAA were identified as the core targets for the treatment of thrombosis.

### Biological function and pathway enrichment analysis

Gene Ontology (GO) and Kyoto Encyclopedia of Genes and Genomes (KEGG) pathway enrichment analyses were used to elucidate the mechanisms through bio-logical processes (BP), cellular components (CC), molecular functions (MF), and key signaling pathways using the Metascape system database (http://metascape.org/, up-dated September 16, 2020) [21]. The species was focused on “Homo sapiens”, and the enrichment of pathway was considered significant when the modified fisher exact false discovery rate (FDR) was less than 0.01. The GO and KEGG results were visually analyzed by an online bioinformatics platform (http://www.bioinformatics.com.cn/).

### Construction of the C-T-P network

Based on the common targets shared between compounds and diseases and the most highly predicted pathways, a “component-target-pathway” regulatory network was constructed using Cytoscape software.

### Molecular docking analysis

Molecular docking is a widely used computer virtual screening technology for predicting the interaction mode and affinity between a ligand and a receptor that is based on geometric and energy matching principles. In this study, the TCMSP (http://tcmspw.com/tcmsp.php) database was used to obtain the active ingredient in a MOL format file. The file was then imported into the Sybyl-X 2.0 energy optimization software program, and saved in mol2 format for later use. After downloading the PDB format file of the crystal structure of the core target protein from the RSCB PDB database (https://www.rcsb.org/), Sybyl-X 2.0 software was used for a series of optimization operations, such as ligand extraction and hydrodehydration of the target protein. The docking mode of the receptor protein and ligand compound was observed using the Surflex-Dock GeomX module in the software program. Discovery Studio was used to visualize and analyze the docked conformations. Afterwards, by comparing the docking score with the original ligand, compounds with a higher value were screened out.

Ten target proteins were chosen in our investigation, including: ALB (Albumin, PDB ID: 7X7X), SRC (Proto-oncogene tyrosine-protein kinase Src, PDB ID: 2BDF), MMP9 (Matrix metalloproteinase-9,PDB ID: 6ESM), CASP3 (Caspase-3,PDB ID: 1RHU), EGFR (Epidermal growth factor receptor, PDB ID: 7ZYM), FGF2 (Fibroblast growth factor 2, PDB ID: 2FGF), KDR (Vascular endothelial growth factor receptor 2, PDB ID: 6GQQ), MMP2 (Matrix metalloproteinase-2, PDB ID: 8H78), F2 (Thrombin, PDB ID: 1DWC), and F10 (Coagulation factor Xa, PDB ID: 1XKA).

### Determination of gene expression level changes using qRT-PCR

To elucidate the molecular effects of DS-CS on zebrafish, the qPCR experiment was carried out with an ABI QuantStudioTM 7 Flex Real-Time PCR System (CA, USA). The total RNA of zebrafish was extracted by RNAiso Plus reagent and the RNA concentration was measured by NanoDrop 2000 spectrophotometer (CA, USA). Then, the RNA reverse transcription was performed with Primescript RT master mix (TaKaRa, China) to synthesize cDNA. According to the manufacturer’s protocol, the final PCR reaction system was 10 μL, including 5 μL HieffTM qPCR SYBR^®^ Green Master Mix, 0.2 μL PCR reverse primer, 0.2 μL PCR forward primer, 2 μL template cDNA, and 2.6 μL ddH_2_O. The reaction conditions of PCR were as follows: 95 °C for 5 min for initial denaturation, then 40 cycles of denaturation at 95 °C for 10 s, annealing and extension at 60 °C for 30 s. β-actin was regarded as the reference gene and the 2^−ΔΔCT^ method was carried out to analyze the relative mRNA expressions (Table 1). Each experiment was conducted in triplicate.

**Table 1:** Primer sequences for RT-qPCR analysis in the zebrafish

Gene	Forward primer (5′-3′)	Reverse primer (5′-3′)
*EGFR*	ACGCAGACGAGTATTTAGTGCCCA	AGTTTCCAAAGCTGCTGTTCAGGC
*F10*	ATGTGGTCTGCTCGTGTGCTAATG	TGTTCGGCGTGTGGAAGAAGATG
*SRC*	TGTTGGATAATGAACGGACGC	TAGCGTTCAGATGCAGACCC
*β-actin*	CCCCATTGAGCACGGTATTG	ATACATGGCAGGGGTGTTGA

### Statistical analysis

All data were expressed as the mean ± standard deviations (SD) of three different experiments. Multiple group comparison was conducted by one-way analysis of variance (ANOVA) of IBM SPSS Statistics 19. A *p*-value of less than 0.05 was considered as statistically significant.

## Results

### Determination of PF and SAB in DS-CS extract

**Fig. 1:**
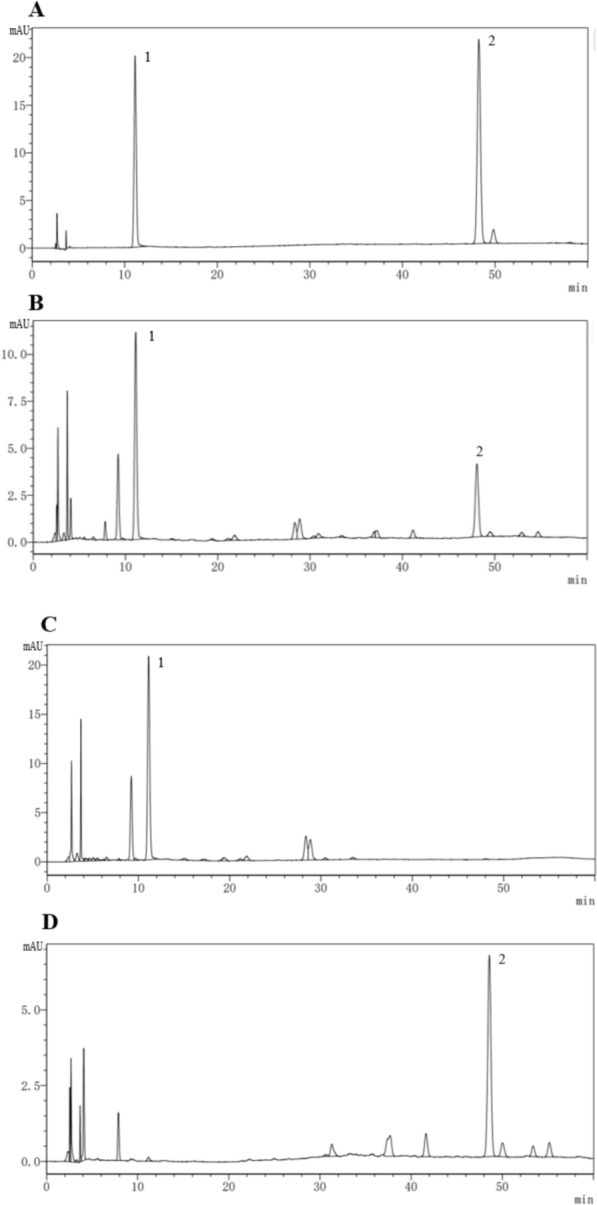
The HPLC chromatograms of **A** mixture of reference compounds; **B** DS: CS = 1:1; **C** CS; and **D** DS. 1, Paeoniflorin; 2, salvianolic acid B

In accordance with the Pharmacopoeia of the People's Republic of China, this study selected PF and Salvianolic Acid B (SAB) as the primary indicators for the quality assessment of the DS and CS formula granules. PF and SAB in the DS-CS extract were quantitatively determined and the results were shown in Table 2. The HPLC chromatograms of the mixed standard of PF and SAB, DS granules, CS granules, and the mixture of the two granules at the ratio of 1:1 (DS-CS extract) were shown in Fig. 1. This method can provide a reference for the quantification of the content in mixtures of DS formula granules and CS formula granules.

**Table 2:** the contents of PF and SAB in DS-CS extract

sample	Paeoniflorin (mg/g)	salvianolic acid B (mg/g)
DS:CS (1:1)	83.20	37.06
DS:CS (0:1)	84.53	
DS:CS (1:0)		37.45

### Evaluation of the survival and hatching rates of zebrafish following treatment with DS-CS extract, PF, and SAA

We evaluated the effects of DS-CS extract on the survival and hatching rates of zebrafish. It was found that the survival rate of zebrafish demonstrated a dose-dependent decrease as the drug concentration increased from 100 μg/mL to 400 μg/mL and the exposure time extended from 24 h post-fertilization (hpf) to 96 hpf. Particularly, at a concentration of 400 μg/mL from 72 hpf onwards, the survival rate significantly decreased compared to the control group (*p* < 0.05). However, at concentrations of 100 μg/mL and 200 μg/mL, the survival rates of zebrafish were not significantly different from the control group. Furthermore, a concentration of 400 μg/mL significantly inhibited zebrafish hatching, whereas concentrations of 100 and 200 μg/mL had no significant effect on the hatching rate.

**Fig. 2:**
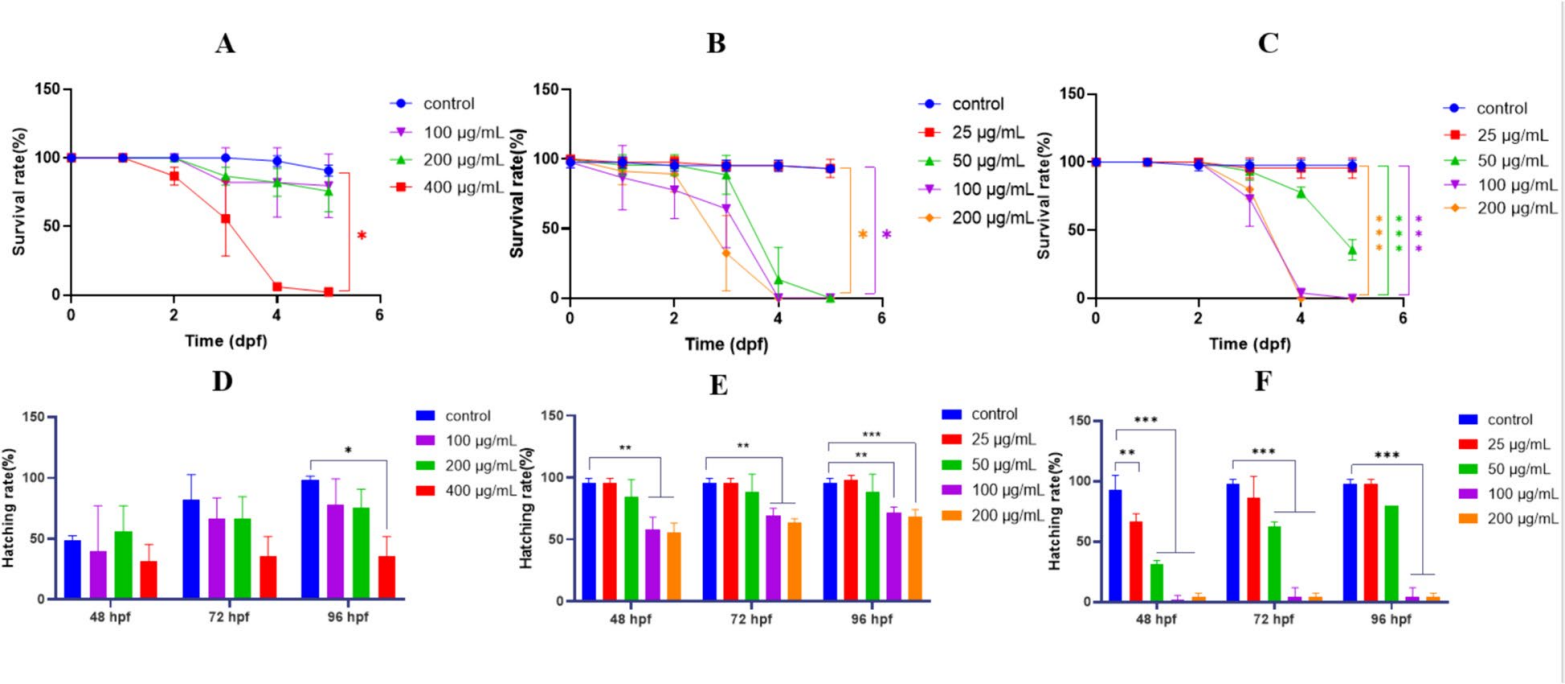
**A** survival rate of DS-CS extract (100, 200 and 400 μg/mL) treatment group; **B** survival rate of PF (25, 50, 100, and 200 μg/mL) treatment group; **C** survival rate of SAA (25, 50, 100, and 200 μg/mL) treatment group; **D** hatching rate of DS-CS extract (100, 200 and 400 μg/mL) treatment group; **E** hatching rate of PF (25, 50, 100, and 200 μg/mL) treatment group; **F** hatching rate of SAA (25, 50, 100, and 200 μg/mL) treatment group. *p < 0.05, **p < 0.01 and***p < 0.001 versus the control group

Subsequently, we assessed the effects of SAA and PF on the survival and hatching rates of zebrafish. The results indicated that at concentrations of 25 and 50 μg/mL for PF and 25 μg/mL for SAA, the survival rates of zebrafish were not significantly different from the control group. At concentrations of 100 and 200 μg/mL, the PF treatment group showed significant differences in hatching rates compared to the control group at 48, 72, and 96 h post-fertilization (hpf). Additionally, SAA markedly inhibited the development of zebrafish at the same concentrations (100 and 200 μg/mL). These findings indicate that both higher concentrations of PF and SAA significantly affect the hatching rates of zebrafish (Fig. 2).

### Assessment of the antithrombotic effect of DS-CS extract in a PHZ-induced zebrafish thrombosis model

**Fig. 3:**
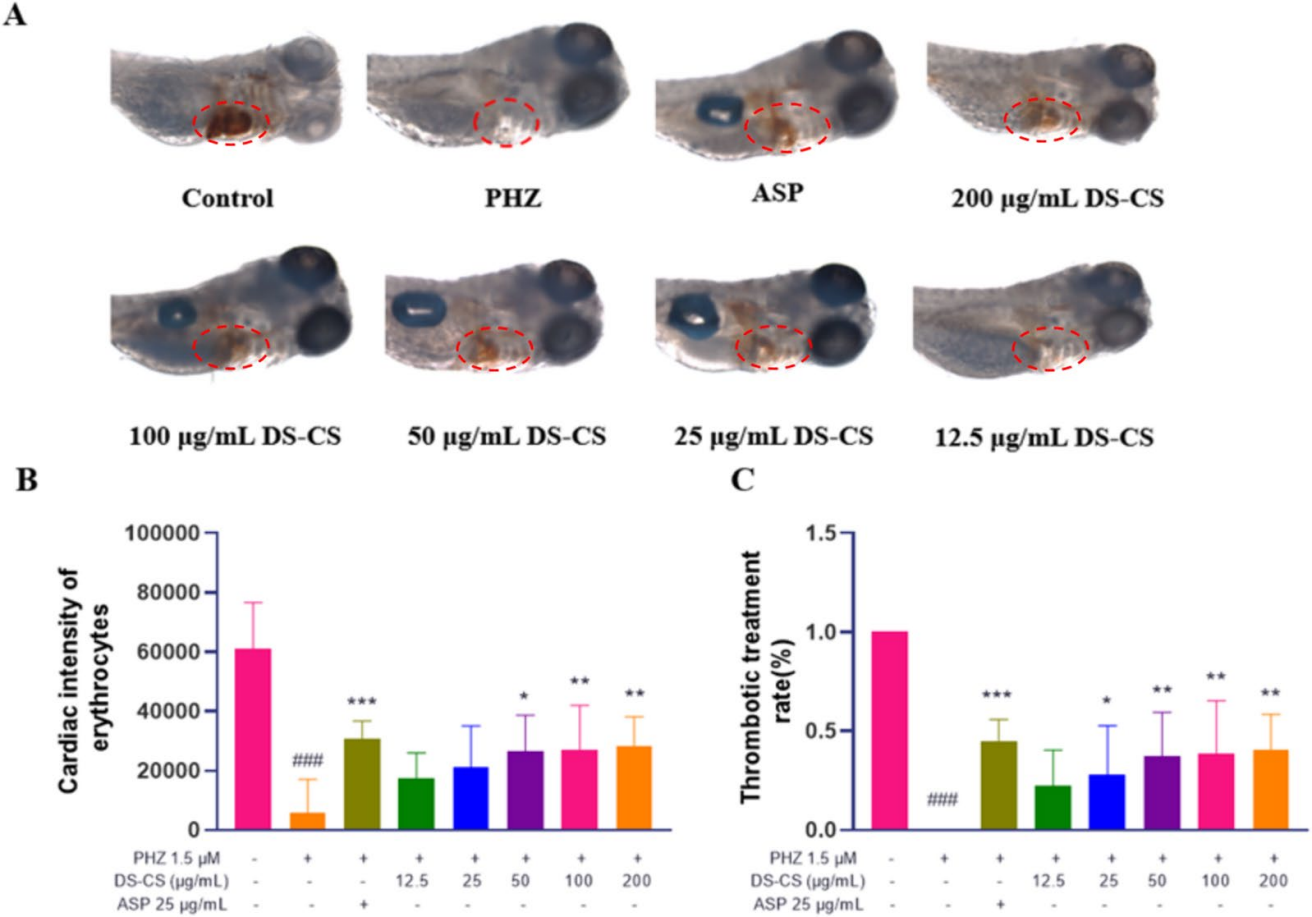
The intensity of cardiac erythrocytes (**A**), thrombotic inhibition percentage (**B**), and the thrombus staining area in the heart (**C**) of zebrafish larvae. ^###^*p* < 0.001 versus the control group, *p < 0.05, **p < 0.01 and ***p < 0.001 versus the model group (PHZ)

The antithrombotic potential of DS-CS extract was assessed using an in vivo zebrafish thrombosis model, with the results depicted in Fig. 3B. The thrombotic inhibition percentages for the 25, 50, 100, 200, 300 μg/mL DS-CS extract treatment groups were calculated as 28% (*P* < 0.05), 37% (*P* < 0.001), 39% (*P* < 0.001), 40% (*P* < 0.001) and 42% (*P* < 0.001) respectively, indicating the therapeutic effect of DS-CS extract in PHZ-induced zebrafish thrombosis model (Fig. 3C). The antithrombotic effect was further corroborated through microscopic examination, revealing the preservation of a dark red coloration within the cardiac region (Fig. 3A). This visual confirmation underscores the anti-thrombotic properties of DS-CS extract.

### Assessment of the antithrombotic effect of PF and SAA on PHZ-induced zebrafish thrombosis model

**Fig. 4:**
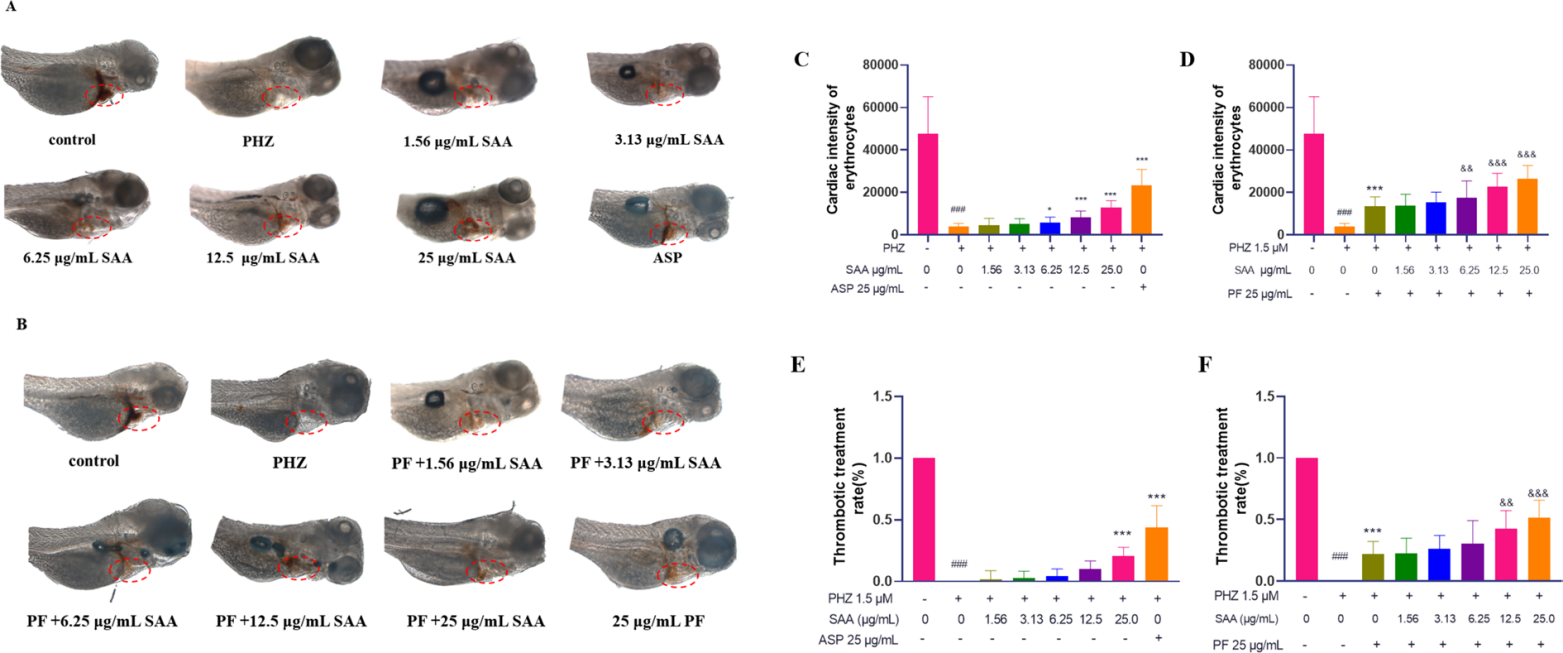
The area of heart thrombus staining in zebrafish was treated with SAA alone (**A**) or combined with PF (**B**). Zebrafish cardiac erythrocyte intensity was treated with SAA alone (**C**) or combined with PF (**D**). **E** The rate of thrombotic treatment with SAA alone; **F** Rate of thrombosis in SAA combined with PF. Statistical significance is denoted as follows: *p < 0.05 and ***p < 0.001 versus the PHZ-induced model group; ^###^*p* < 0.001 versus the control group; ^&&^*p* < 0.01, ^&&&^*p* < 0.001 versus the group treated with PF at 25 μg/mL

Figure 4 illustrated the antithrombotic activities of PF at a concentration of 25 μg/mL, SAA at concentrations of 0, 1.56, 3.13, 6.25, 12.5, and 25 μg/mL, as well as their combinations in 48 hpf zebrafish. Compared to the model group (concentrations of PF and SAA are 0 μg/mL), SAA monotherapy showed significant differences at concentrations of 6.25, 12.5 and 25 μg/mL in a dose dependent manner. PF demonstrated a substantial rescuing effect on PHZ-induced cardiac erythrocyte reduction at a concentration of 25 μg/mL, whether administered alone or in combination with SAA at varying concentrations. Notably, the combination treatment approach also displayed a dose-dependent response as the concentration of SAA increased from 1.56 to 25 μg/mL, while PF remained constant at 25 μg/mL. The most favorable rescuing effect was observed when both PF and SAA were administered at concentrations of 25 μg/mL, with a 1:1 ratio between the two compounds.

The results of the combination therapy of PF and SAA in the treatment of thrombosis indicate a significant difference in efficacy compared to the PHZ group, with the thrombotic effects of SAA and PF alone being 21% and 34%, respectively. When PF and SAA were administered in combination at concentrations of 25 μg/mL + 25 μg/mL and 25 μg/mL + 12.5 μg/mL, the therapeutic effects on thrombosis improved to 43% and 51%, respectively, showing a significant enhancement in efficiency compared to the group treated with PF alone. Furthermore, the anti-thrombotic efficiency of the combined application of PF and SAA was very close to that of the DS-CS extract group, suggesting that PF and SAA may be the key active components in DS-CS.

### Identification of potential target genes associated with DS-CS and thrombosis

Chemical constituents from DS and CS were retrieved using the Traditional Chinese Medicine Systems Pharmacology (TCMSP) database (http://tcmspw.com/tcmsp.php). Potential target genes for PF and SAA were obtained through PharmMapper and Swiss Target Prediction databases. Following UniProt standardization and deduplication, a total of 149 potential targets were identified for PF and SAA.

To enrich thrombosis-related targets, a keyword search for "Thrombosis" was conducted, yielding 1123 thrombosis-related targets from GeneCards. Additionally, 34 targets were obtained from DrugBank, 63 from PharmGKB, and 11 from the OMIM database. After eliminating duplicates, a comprehensive set of 1177 thrombosis-related targets were assembled for further analysis.

### Identification of drug–disease intersection targets

**Fig.5:**
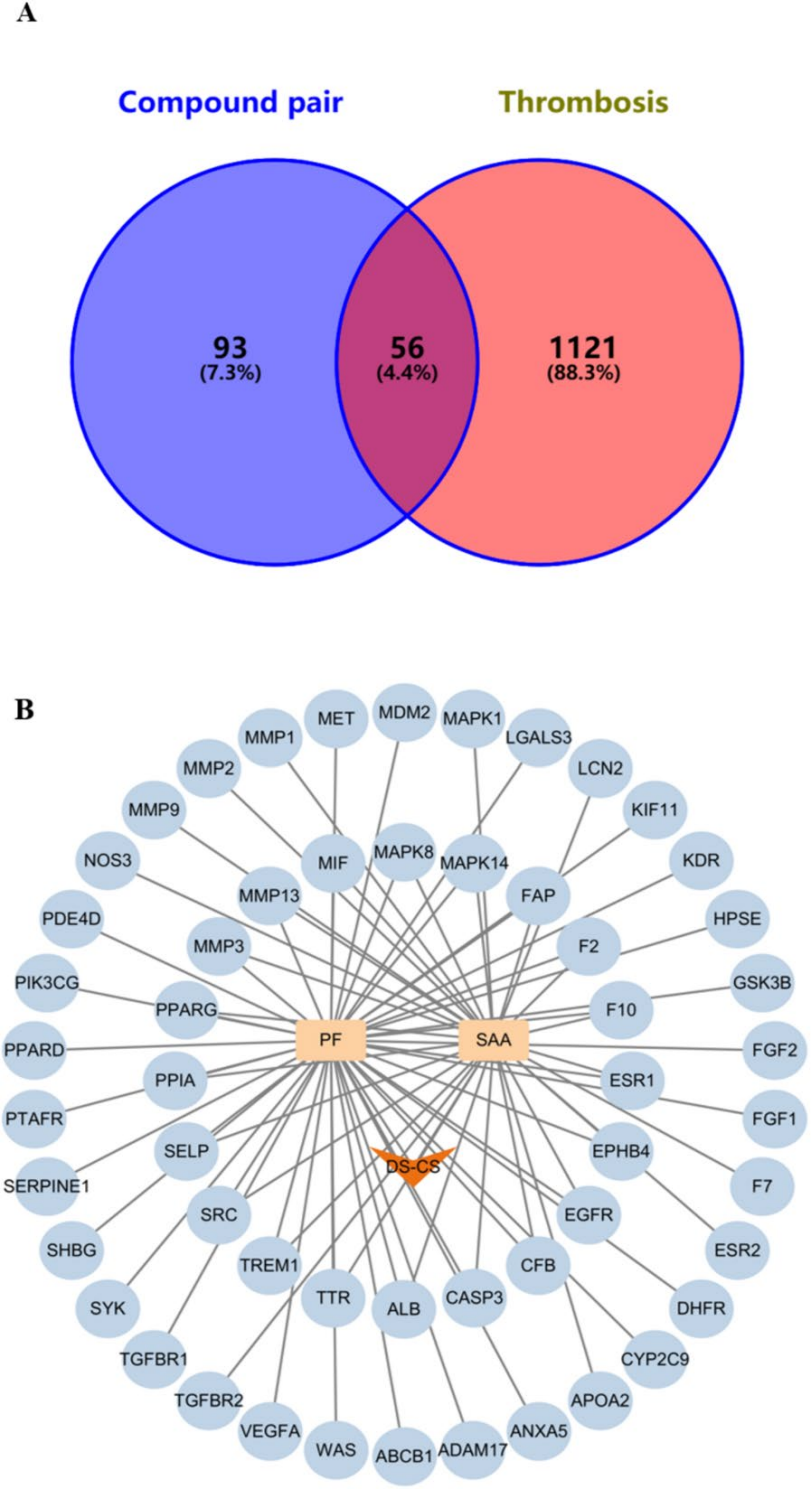
**A**Venn diagram of the intersection targets of drug and thrombosis; **B** Drug target interaction diagram

A Venn analysis was conducted, employing the 149 potential targets of PF and SAA, in conjunction with the 1177 thrombosis-related target genes. This analysis revealed 56 drug–disease intersection gene targets, as depicted in Fig. 5, which were subsequently subjected to further analysis.

### Protein–protein interaction (PPI) network analysis

**Fig. 6:**
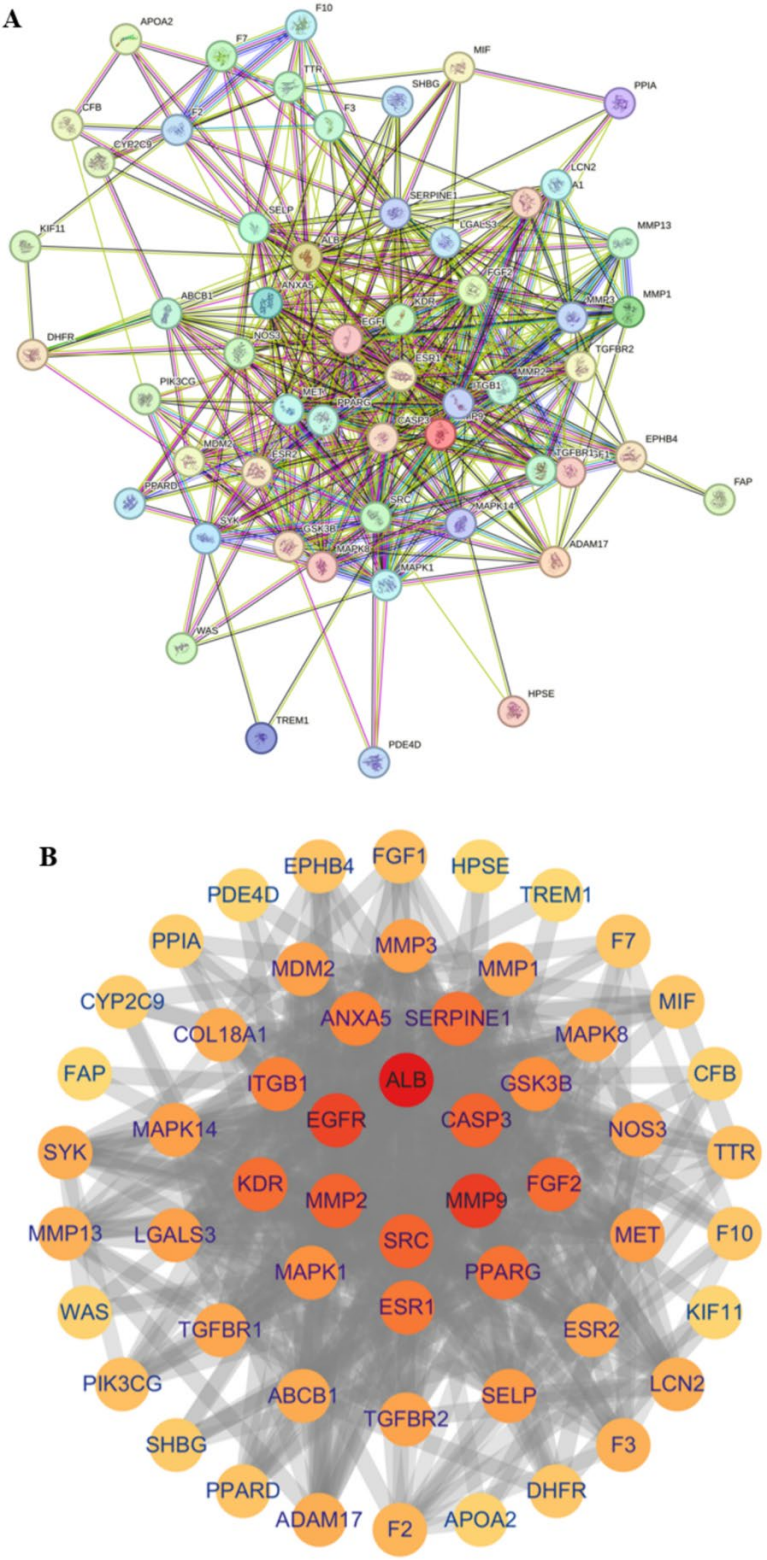
**A** PPI network of targets generated using STRING 11.0; **B** Potential targets were arranged according to the degree value from large to small

The 56 drug–disease intersection gene targets were analyzed using a PPI network constructed with STRING database, as shown in Fig. 6A. The network was com-posed of 56 nodes and 446 edges, and the average node degree was 15.9, with a PPI enrichment *P*-value of < 0.05. The results of STRING analysis were im-ported into Cytoscape software. The network analysis plug-in was used to count the nodes in the network graph and analyze their connectivity according to the node degree. A higher node degree within the network corresponded to a greater number of biological functions associated with that node. The result of PPI analysis revealed that the therapeutic targets of PF and SAA exhibit a distinct feature of multifaceted net-works and synergistic interactions. The network was constructed as shown in Fig. 6B. The ten most-connected targets were ALB, SRC, MMP9, EGFR, FGF2, KDR, MMP2, F2, and F10.

### GO and KEGG pathway enrichment analysis

A comprehensive set of 828 significantly enriched Gene Ontology (GO) entries was obtained through Metascape analysis, encompassing 714 Biological Process (BP), 76 Molecular Function (MF), and 38 Cellular Component (CC) categories. Within the BP category, predominant themes included angiogenesis, blood coagulation, and regulation of angiogenesis. In the CC category, extracellular matrix, external encapsulating structure, and vesicle lumen were prominent, while the MF category featured serine-type endopeptidase activity, protein kinase activity, and peptidase activity.

**Fig. 7:**
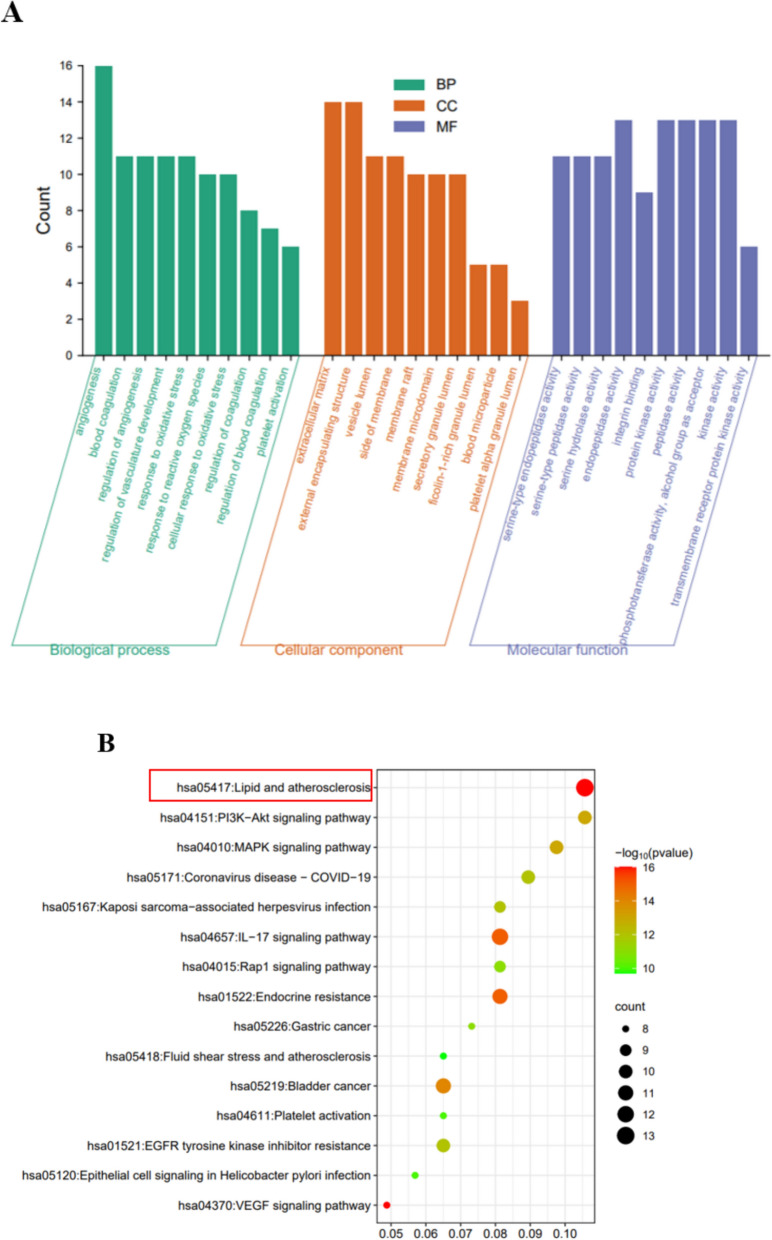
**A** GO enrichment analysis depicting the top 10 BP, MF, and CC categories; **B** Bubble chart illustrating the top 15 KEGG pathways

These findings suggest that PF and SAA may exert antithrombotic effects through the modulation of metabolic processes, inflammatory factors, cell proliferation, protein transport, transcription factor activity, and other biological processes. Further-more, our analysis identified a total of 123 enriched pathways, with the most highly enriched pathways encompassing lipid metabolism, atherosclerosis, fluid shear stress, PI3K-Akt signaling, and VEGF signaling. These pathways are primarily associated with inflammation, vasculogenesis, immunity, hormone regulation, among others. The top 10 GO entries and top 15 Kyoto Encyclopedia of Genes and Genomes (KEGG) pathways, based on FDR and hit gene counts, are presented in Fig. 7A, B for reference.

### Construction and analysis of the C-T-P network for PF and SAA in thrombosis

**Fig. 8:**
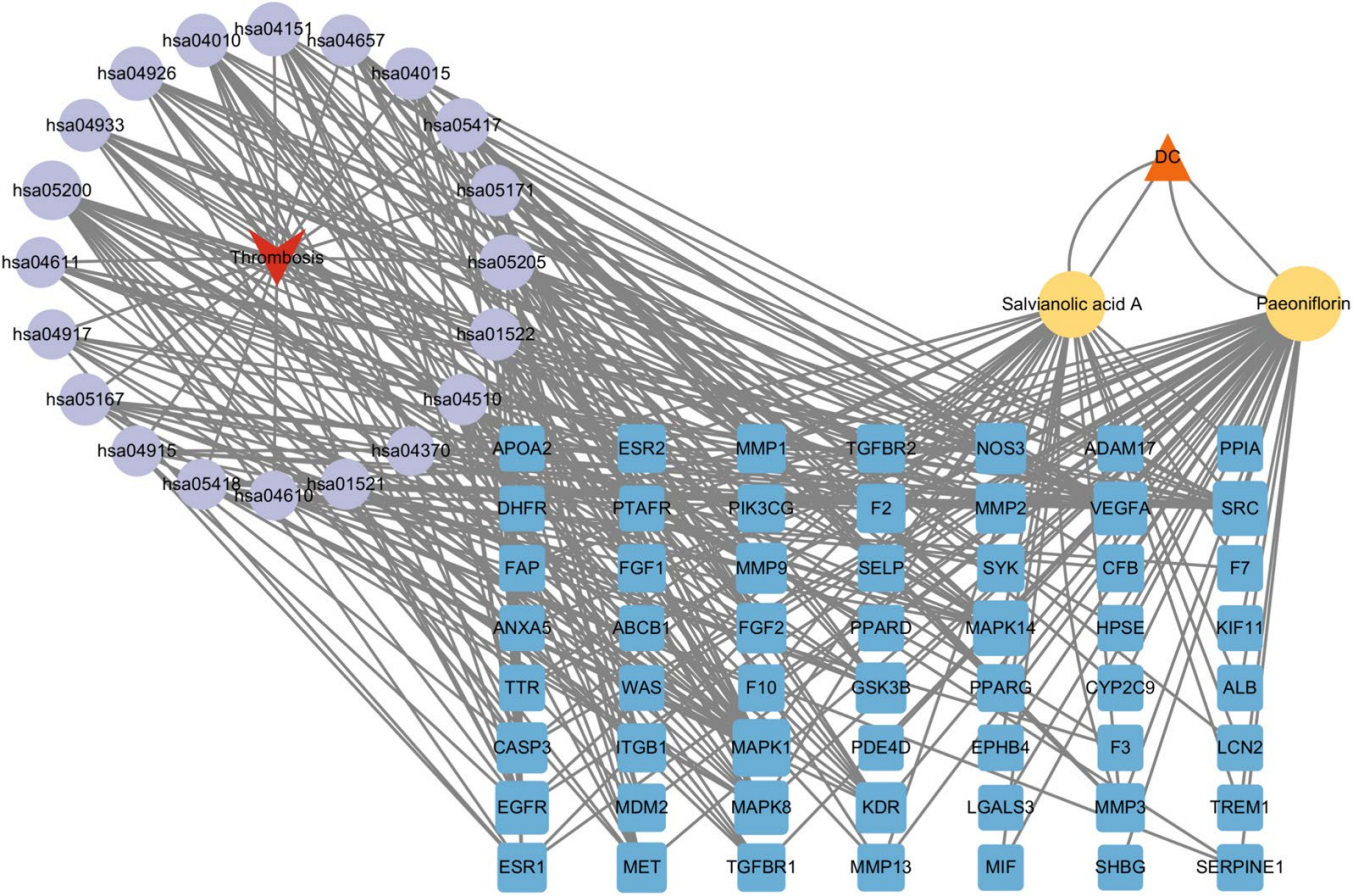
C-T-P Network for PF and SAA in Thrombosis

To reveal the intricate multi-component and multi-target effects of PF and SAA in the management of thrombosis and gain insights into their mechanisms of action, a compound-target-pathway (C-T-P) network was constructed and analyzed. This net-work was composed of 80 nodes and 306 edges as shown in Fig. 8. The assessment of network topological parameters aids in the identification of pivotal nodes, encompassing compounds and targets that assume significant roles within the network. In this context, node degree was employed to discern essential components and core targets. Notably, PF and SAA demonstrated the capacity to concurrently influence multiple targets, while certain targets were susceptible to modulation by multiple compounds concurrently.

### Molecular docking analysis

Molecular docking analyses revealed the docking scores for both PF and SAA with a panel of target proteins, including ALB, SRC, MMP9, CASP3, EGFR, FGF2, KDR, MMP2, F2, and F10, indicative of the binding affinity (Table 3). Interaction diagrams for the top three highest-scoring docking conformations are presented herein, with de-tailed hydrogen bond and amino acid residue interactions summarized in Tables 4 and 5.

**Table 3:** Docking scores of active compounds of PF and SAA with core targets

Compound	Molecular ID	Targets	PDB ID	Total score	CSCORE
Paeoniflorin	MOL0071924	SRC	2BDF	10.99	4
		EGFR	7ZYM	10.47	4
		F10	1XKA	10.35	4
		ALB	7X7X	9.32	5
		MMP2	8H78	8.78	4
		KDR	6GQQ	7.99	2
		CASP3	1RHU	7.76	5
		F2	1DWC	7.58	5
		MMP9	6ESM	6.3298	5
		FGF2	2FGF	4.41	4
Salvianolic acid A	MOL007136	EGFR	7ZYM	9.44	3
		F10	1XKA	9.35	5
		SRC	2BDF	8.9443	4
		MMP9	6ESM	8.57	5
		KDR	6GQQ	8.27	4
		CASP3	1RHU	7.86	5
		MMP2	8H78	7.34	5
		ALB	7X7X	7.22	4
		F2	1DWC	6.74	4
		FGF2	2FGF	4.75	4

**Table 4:** Docking results of investigated SAA with EGFR、F10、SRC

Target proteins	HB	Other amino acid residues
EGFR	GLN791, CYS775, MET790, ASP855, LYS745, MET793, ASP800	MET766, THR854, ALA743, ASN842, LEU844, PHE795, LEU792, PHE723, VAL726, GLY796, VAL845, LEU718, SER797, GLY719
F10	ARG143, GLN192, GLU146, LYS147, GLY218, ASP189, ALA190, SER195, SER214 GLN61	ARG222, CYS220, GLY216, GLY226, ILE227, CYS191, ASP194, VAL213, TRP215, TYR99, HIS57
SRC	ASP404, ASN391, LYS295, PHE278, ASP348, LEU273, MET341, CYS277	ALA403, ALA390, GLY279, GLY276, GLU280, SER345, GLN275, GLY274, GLY344, THR338, LEU393, ALA293, VAL281, GLU339, TYR340

**Table 5:** Docking results of investigated PF with SRC、EGFR、F10

Target proteins	HB	Other amino acid residues
SRC	ASP348, THR338, MET341, GLU339	PHE307, PHE278, GLY279, LYS295ASP404, VAL281, SER345, LEU273, GLY344, GLY276, GLU280, GLN275, GLY274, LEU393, VAL323, ALA293, TYR340
EGFR	ASP800, SER797, THR854, LYS745, MET793	GLY719, ARG841, ASN842, GLY796, ASP855, LEU844, VAL726, CYS775, MET790, GLN791, ALA743, LEU1001, PRO794, PHE795, LEU792, PHE723
F10	GLN61, GLN192, TYR99, LYS96, GLU97	THR98, PHE174, GLU217, SER195, SER214, GLY193, VAL213, ASP194, GLY218, CYS220, CYS191, ALA190, TRP215, HIS57, GLY226, ASP189

**Fig. 9:**
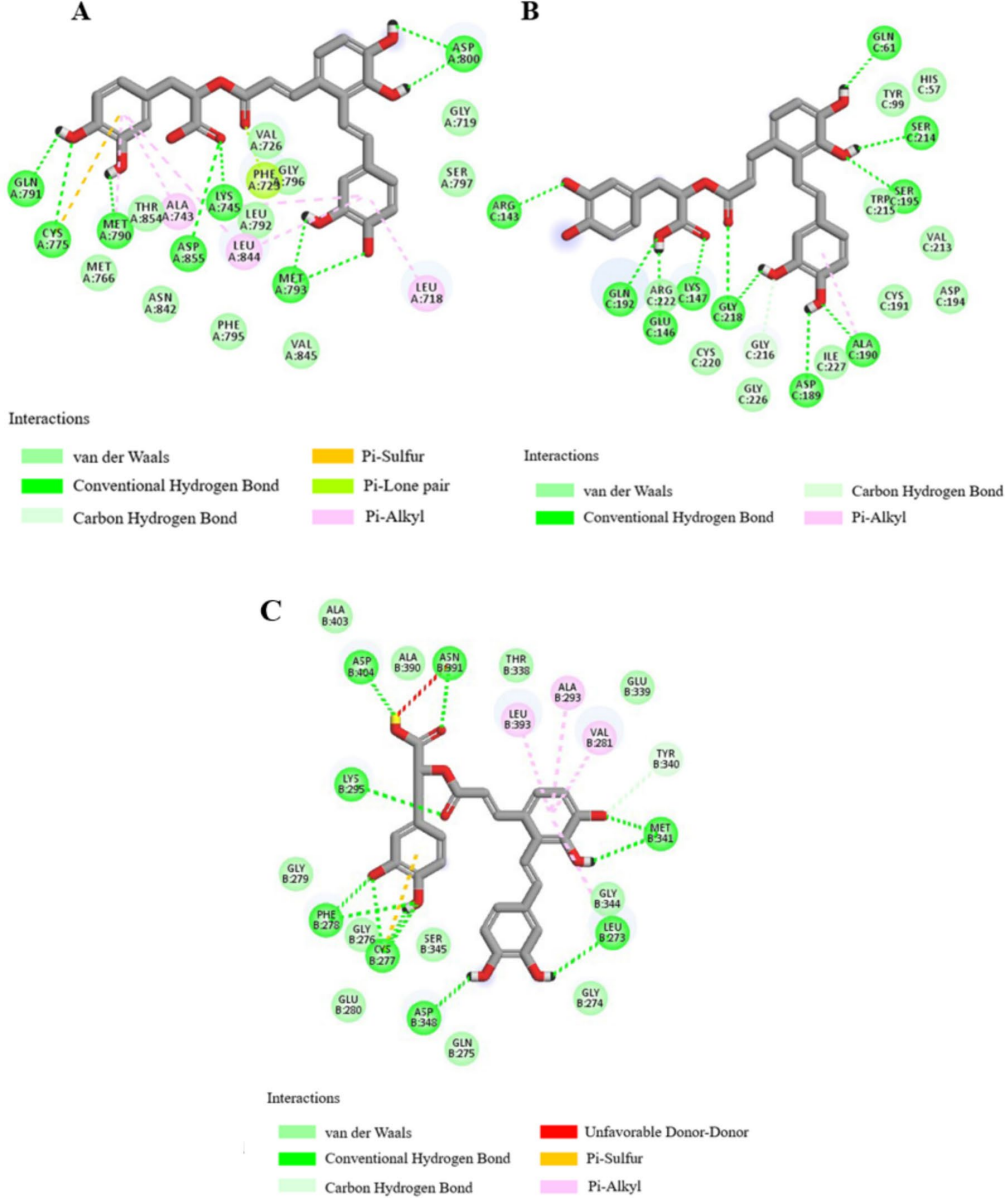
Docking results of the compounds SAA with EGFR (PDB ID: 7ZYM) (**A**), F10 (PDB ID: 1XKA) (**B**), SRC (PDB ID: 2BDF) (**C**)

For SAA, robust binding interactions were observed with EGFR, F10, SRC. Specifically, SAA exhibited stable binding to EGFR through interactions with GLN791, CYS775, MET790, ASP855, LYS745, MET793, and ASP800 (Fig. 9A). Within the active site of F10, SAA established hydrogen bonding interactions with ARG143, GLN192, GLU146, LYS147, GLY218, ASP189, ALA190, SER195, SER214, and GLN61 on the F10 target protein (Fig. 9B). Interaction with SRC involved hydrogen bonds with ASP404, ASN391, LYS295, PHE278, ASP348, LEU273, MET341, and CYS277 (Fig. 9C).

**Fig. 10:**
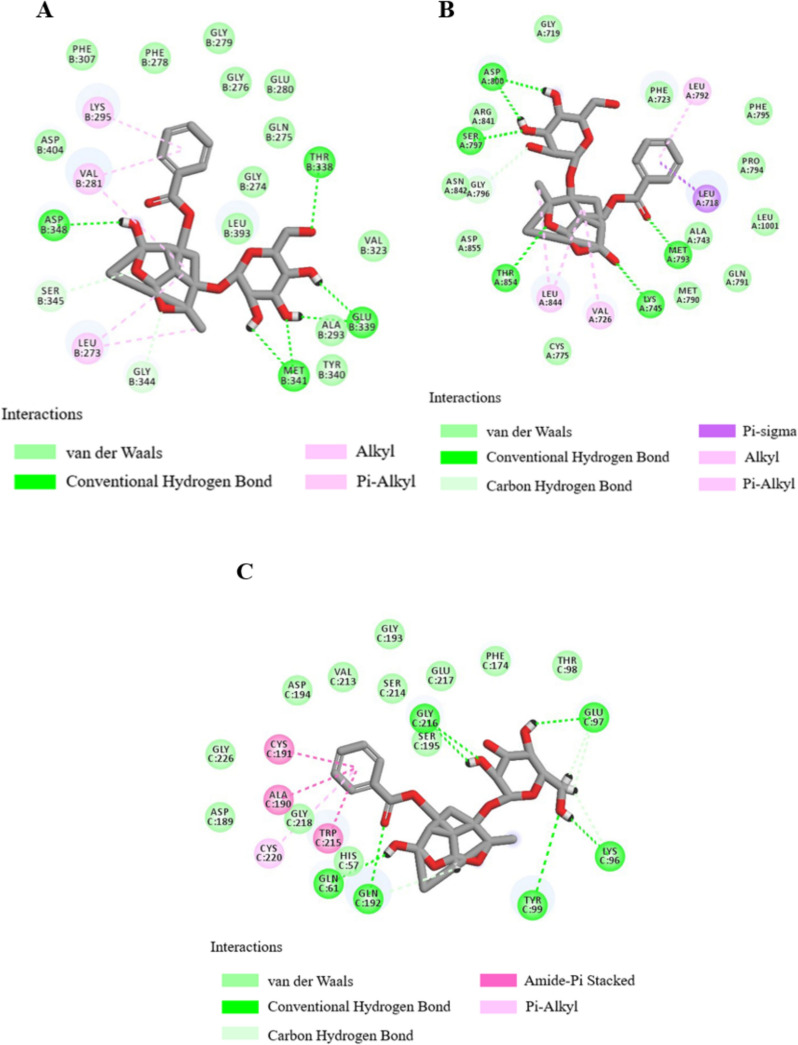
Docking results of the compounds PF with SRC (PDB ID: 2BDF) (**A**), EGFR (PDB ID: 7ZYM) (**B**), F10 (PDB ID: 1XKA) (**C**)

Similarly, PF demonstrated robust binding to SRC, EGFR, F10. PF established stable binding to SRC through interactions with ASP348, THR338, MET341, and GLU339 on the SRC target protein (Fig. 10A). Within the active site of EGFR, PF engaged in hydrogen bonding interactions with ASP800, SER797, THR854, LYS745, and MET793 (Fig. 10B). Binding to F10 was characterized by hydrogen bonds with GLN61, GLN192, TYR99, LYS96, and GLU97 (Fig. 10C).

### The mRNA expression levels of DS-CS in zebrafish

**Fig. 11:**
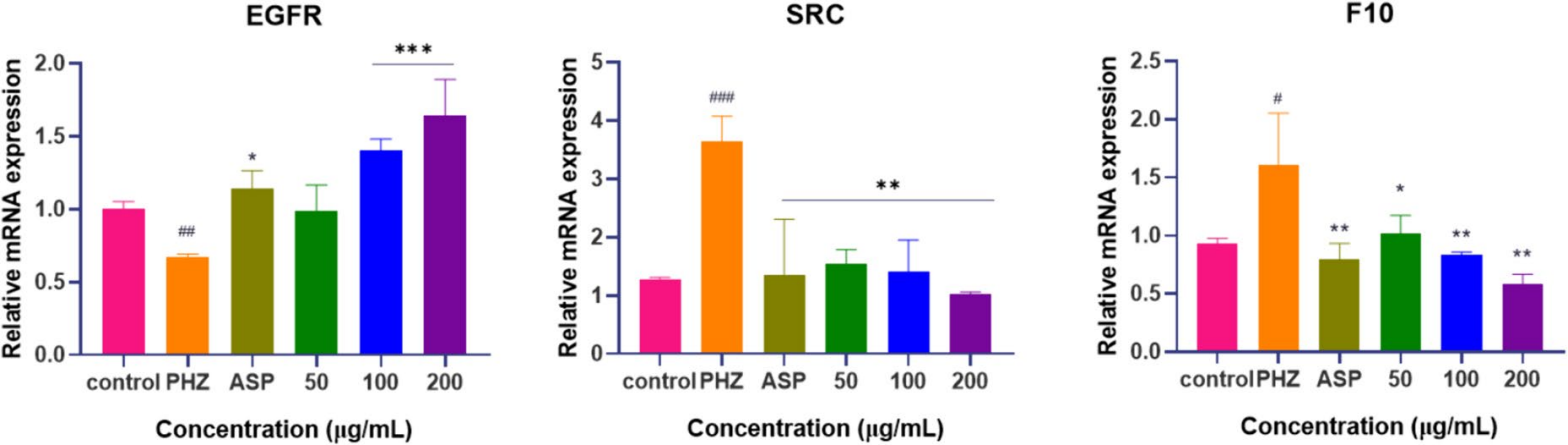
mRNA expression levels of EGFR, SRC and F10 induced by DS-CS

In order to validate the three target proteins screened from molecular docking analysis, mRNA expression levels of EGFR, SRC, and F10 in zebrafish (Fig. 11) were investigated. Zebrafish tissues mRNA was extracted and subjected to DS-CS intervention across varying concentrations. Through qRT-PCR analysis, it was revealed that compared to the normal control group, the mRNA expression of EGFR decreased in the PHZ group. DS-CS upregulated the expression levels of EGFR mRNA, and treatment with different concentrations of DS-CS significantly elevated the mRNA levels of the EGFR induced by PHZ in zebrafish (*P* < 0.05). Furthermore, compared to the normal control group, the mRNA expression levels of F10 and SRC increased in the PHZ group. DS-CS downregulated the mRNA levels of F10 and SRC (*P* < 0.05), thereby ameliorating thrombus formation.

## Discussion and conclusion

In this study, we evaluated the pharmacological activities of DS-CS and its active components, SAA and PF, using a phenylhydrazine-induced thrombosis model in zebrafish. DS-CS significantly improved reduced cardiac blood flow induced by PHZ within the concentration range of 12.5 μg/mL to 200 μg/mL, indicating notable anti-thrombotic effects, especially at higher concentrations. Additionally, we investigated the combined anti-thrombotic activity of SAA and PF, observing significant differences compared to single PF treatment. The combined application of PF and SAA at a ratio of 25 μg/mL + 25 μg/mL showed pronounced anti-thrombotic effects, potentially through additive mechanisms. Furthermore, the therapeutic effects of combined PF and SAA administration surpassed those of PF alone, indicating enhanced efficiency.

To elucidate the potential targets engaged by SAA and PF, along with the underlying mechanisms of action, we harnessed the power of network pharmacology. This comprehensive approach pinpointed ten pivotal genes, namely ALB, SRC, MMP9, CASP3, EGFR, FGF2, KDR, MMP2, F2 and F10, which emerged as closely associated with the antithrombotic attributes of PF and SAA. By amalgamating PPI analysis with KEGG pathway exploration, it became ap-parent that SAA and PF predominantly influence pathways intertwined with inflammation, vasculogenesis, immunity, hormonal regulation, and, notably, lipid metabolism and atherosclerosis. Among these ten key target proteins, SRC, EGFR, and F10 exhibited robust binding affinities to PF and SAA, as corroborated by molecular docking studies. The qPCR experimental results indicate that DS-CS upregulates the mRNA expression levels of EGFR while inhibiting the mRNA expression levels of F10 and SRC, thereby ameliorating the condition of thrombus formation.

In summary, our study validates the antithrombotic potential of DS-CS and its constituents, shedding light on the underlying mechanisms. These findings offer insights for potential therapeutic development in thrombotic disorders, emphasizing the significance of DS-CS in clinical applications.

## Data Availability

The datasets used and/or analyzed during the current study are available from the corresponding author on reasonable request.

## Abbreviations

SAA: Salvianolic acid A
PF: Paeoniflorin
ALB: Albumin
SRC: Proto-oncogene tyrosine-protein kinase Src
MMP9: Matrix metalloproteinase-9
EGFR: Epidermal growth factor receptor
FGF2: Fibroblast growth factor 2
KDR: Kinase insert domain receptor
MMP2: Matrix metalloproteinase-2
F2: Prothrombin
F10: Coagulation factor X

## References

[1] Vermeersch E (2017). The role of platelet and endothelial GARP in thrombosis and hemostasis. PLoS ONE.

[2] Stowell SR, Stowell CP (2019). Biologic roles of the ABH and Lewis histo-blood group antigens part II: thrombosis, cardiovascular disease and metabolism. Vox Sang.

[3] Nakanishi M (2016). Emergency cardiac surgery and heparin resistance in a patient with essential thrombocythemia. JA clinical reports.

[4] Stupnisek M, Pentadecapeptide BPC (2012). 157 reduces bleeding time and thrombocytopenia after amputation in rats treated with heparin, warfarin or aspirin. Thromb Res.

[5] Zhou X (2019). Synergistic study of a Danshen (Salvia Miltiorrhizae Radix et Rhizoma) and Sanqi (Notoginseng Radix et Rhizoma) combination on cell survival in EA. hy926 cells. BMC Complement Altern Med.

[6] Zuo HL (2020). Interactions of antithrombotic herbal medicines with Western cardiovascular drugs. Pharmacol Res.

[7] Zhang DY (2022). A network pharmacology-based study on the quality control markers of antithrombotic herbs: Using Salvia miltiorrhiza - Ligusticum chuanxiong as an example. J Ethnopharmacol.

[8] Su CY, Ming QL (2015). Salvia miltiorrhiza: traditional medicinal uses, chemistry, and pharmacology. Chin J Nat Med.

[9] Mo X, Zhao N (2011). The protective effect of peony extract on acute myocardial infarction in rats. Phytomedicine.

[10] Wang Y (2020). Research progress of salviae miltiorrhiza radixet rhizoma related herb pairs for activating blood and resolving stasis. J Chongqing Univ Technol.

[11] Wang S (2012). Compatibility art of traditional Chinese medicine: from the perspective of herb pairs. J Ethnopharmacol.

[12] Goldsmith JR, Jobin C (2012). Think small: zebrafish as a model system of human pathology. J Biomed Biotechnol.

[13] Jagadeeswaran P (2005). Zebrafish: a tool to study hemostasis and thrombosis. Curr Opin Hematol.

[14] Ma D, Zhang J (2011). The identification and characterization of zebrafish hematopoietic stem cells. Blood.

[15] Delvecchio C, Tiefenbach J (2011). The zebrafish: a powerful platform for in vivo, HTS drug discovery. Assay Drug Dev Technol.

[16] Lu S (2020). Generation and application of the zebrafish heg1 mutant as a cardiovascular disease model. Biomolecules.

[17] Hopkins AL (2008). Network pharmacology: the next paradigm in drug discovery. Nat Chem Biol.

[18] Wang X, Wang ZY (2021). TCM network pharmacology: a new trend towards combining computational, experimental and clinical approaches. Chin J Nat Med.

[19] Zhu XY (2016). A Zebrafish thrombosis model for assessing antithrombotic drugs. Zebrafish.

[20] Szklarczyk D (2017). The STRING database in 2017: quality-controlled protein-protein association networks, made broadly accessible. Nucleic Acids Res.

[21] Zhou Y (2019). Metascape provides a biologist-oriented resource for the analysis of systems-level datasets. Nat Commun.

